# 

**Published:** 1871-01

**Authors:** 


### INDEX TO VOLUME 1.

A

Abortion—Chlorate of Potassa in, 176
Digitalis for hemorrhage. 573
Abscess Hepatic—Chloride Ammonium in, ... 663
After Pains ... 676
A Report on the Relations of the Druggist to the Physician, by Dr. H. W. Hosser ... 642
Ascarides—Hypophosphite of Sodo for ... 671
Of mastoid cells ... 257
Aconite—In lenchen and prurigo, 127
Its uses, ... 311
The child's remedy ... 375
After Pains—Citric acid, ... 75
Bromide potassa, ... 108
Morphia and aconite hypodermically ... 498
Ague—Bromide in, ... 169
Air, Water—Sewerage and drainage, ... 84
Albuminuria—Sambucus canadensis in, ... 124
Liquor calsis in, ... 177
Alcoholism—A remedy for, ... 617
Alum—A remedy for croup, ... 16
Amenorrhœa-Apium gravolens in, 176
A formula for, ... 545
Watespepper in ... 680
Anæsthetic Mixture, ... 184
Effects of the phrenols, ... 372
Carbolic acid as a local, 432
A safe, ... 601
Anæstheria—Local, ... 538–599
Anæsthetics, ... 554
Aneurism—Hypodermic use ergotine in, ... 295
Iodide of potass in, ... 479
Anæmia—Lactate of iron in, ... 170
Prescription for, ... 301
Ether Spray to Spine in, .. 671
Anasarca—Dr. Hale's formula in. 658
Analysis—Of hair restoratives, ... 327
Anderson, Dr. R. B.—On after-pains, ... 108
An old remedy for cystitis, ... 22
A new feature of the journal, ... 67
Angel, Dr. I. W.—On chronic dysentery, ... 580
Angina Pectoris—Amyl in, ... 617
Antidote for carbolic acid, ... 23
Antiperiodics, ... 618
Anus—Radical cure for artificial, . 48
Aphonia—Atropid in nervous. . . 362
A pleasant time, ... 390
Apthæ—Ttreatment of, ... 114
Arnica—New uses of, ... 356
Artificial respiration, ... 619
Arsenic—In uterine affections, 124–171
In pain of stomach and bowels, ... 360
Uses of, ... 497
Ascarides—Santonine for, ... 73
Ascites—Treated by copaiva, ... 314
Asthmatic Bronchitis—Chloral in, 119
Asthma—Treatment of, ... 17
Atomized medication for, 65
Treatment of, ... 57
A remedy for, ... 258
A mixture for, ... 479
Capsicum in, ... 489
Hay...Quinine in ... 592
A specific for, ... 555
Asafœtida—In hooping-cough, ... 15
In epilepsy, ... 296
A quarrel ended, ... 567

B

Bad Breath—Cause and remedy, 87
Battey, Dr. Robert—Catheterism in the female, ... 109
On enemata, ... 405
On coma, ... 520
Intestinal obstructions, .. 637
Baldness—Shampoo for, ... 373
Balsam Peru—In itch, ... 61
Belladonna—In arresting lacteal secretion, ... 73
In infantile icterus, ... 74
In incontenence of urine, . 123
Solution, ... 131
In typoid fever, ... 239
Locally in diabetes, ... 362
Bath—In fever, ... 365
Benzoated ointment, ... 440
Biliary Calculi—A remedy for. ... 684
Bladder, Perchloride of in Paralysis of ... 664
Bromide of Lithium ... 674
Billious colic, ... 121
Billiousness—Nux vomica for, ... 295
Biographical, ... 78
Brantley. Dr. A. H.—Chlo. potass. Is it a parturifacient? ... 526
Boils—Sumac poultices for, ... 476

Branham, Dr. J.—On cholera infantum, .......... 460
Bright's Disease—Lime-water in,. 241
Iodide of potass. in,. 252–368
Nitric acid in,...... 256–429
Ulcers a cause of,........ 369
Bromine Ammonium—In menorrhagia,.......... 21
Potassium in puerperal convulsions, .......... 22
For infants............ 75
In after-pains,.......... 108
In nausea of opium,..... 116
In dysmenorrhœa, ...... 125
In ague,............... 196
Corrective over opium,.. 308
Potassium — In nettle-rash, ...... ........ 310
Bromide Lithium..In epilesy....... 26
Uses of,.................. 60
Bromide Quinine — Morphia and Strychnia.... .......... 428
Bronchitis Chronic—Tonics in,..... 75
A prescription for,......... 129
Hypophosphite of Potass in, 496
Capillary Treatment of,.... 613
Bronchial—Suppressed Secretion,.. 76
Broncocele—Ungent for,........... 127
Broncorrhœa—Tar water in,....... 490
Brinsmades—Diaphoretic,.......... 69
Burns—Chloride of Soda in,........ 16
And Scalds,.............. 125
Internal Complications from, 256
Local Applications for,..... 302
Poultice of Tea leaves to,.. 306
And Scalds, Treatment of,.. 309
And their Treatment,...... 536
White Lead and Flax-seed Oil in,................ 560
Treatment of Sulphurative Stage ................ 683
Buboes..Subcutaneous Injection of, 120
Rapid Cure for,........... 243
Treatment of,............. 490
Bursæ..Treatment of,............. 255

### C

Calabar Bean..In Tetanus,........ 73
In Trismus,.............. 246
Calendula Officinalis..Marigold,.... 71
Camphor..Its Uses,............... 311
Cancer..Application for,........... 246
Chloral in,................ 434
Fluid Extract of Clover in,. 498
Poke Root in,............. 564
Carbolic Acid for.......... 675
Bromide and Kerosene in... 677
Chlorate of Potass. in...... 681
Os Uteri..Treated by Bromine .................. 655
Cancrum Oris..Treatment of,...... 16
Cappillary Bronchitis,............. 439
Carbolic Acid..In Sickness of Pregnancy, ............... 23
In Snake-bite.... ........ 23
Antidote for Poisoning of,.. 23
In Gleet,................ 26
In Eczema,....... ....... 68
In Rubeola,.............. 74
Local use in Phthisis,...... 118
In Diptheria,............. 118
In Uterine Catarrh,........ 125
Use of,.................136–675
In Diphtheretic Affections.. 675
In Hemorrhoids,........... 146
Its Anæsthetic properties,.. 175
In Carbuncles,............ 176
In Pruritus,.............. 255
Deodorized,.............. 257
In Scarlet Fever,....... . 287
In Flavus and Crustea Lactea,..................... 310
In Phthisis,............... 362
In Inhalation,............ 362
As a local Anæsthetic,..... 432
Cerate, .................. 433
Formulæ,................. 484
In Childrens' Diseases,..... 549
Carbolated Alcohol,............... 68
Carbuncle .......................... 238
Treatment of..........471–482
Sulphite of Soda in,....... 485
Calcium..Iodide of............... 677
Calculus Nephritic..A formula for.. 679
Carminative Mixture...Soda Mint,.. 432
Castor Oil..Made palatable,........ 435
Catarrhus Vesicœ..A prescription for,.................. 56
A Formula for............ 636
Catarrh, Common .. A prescription for, .................. 126
Chronic, ................. 171
Senile, Cannabis Indicæ in. 191
Catarrh, Chronic..Aconite in,...... 254
Of Stomach, Nux Vomica in, 314
Nasal, a case of,........... 591
A Snuff for............... 665
Chronic..Intestinal... .... 670
Catarrhal Pneumonia of Infants.. Carb. Ammonia in,......... 354
Cerebro-Spinal Meningitis..By Dr. W. T. Goldsmith,.......... 1
Chloral in,............... 23
Cases by Dr. T. S. Powell,.. 101
Cervix Uteri..Arsenic in Enlargement of,................. 483
Chalk Mixture..Improved formula,. 240
Chancre..Iodoform to,....... .... 182
Camphor in,............. 251
Chapped Lips..................... 564
Character ...... ................. 502
Chesnut-leaves..In hooping Cough,. 24
Cherry Pectoral..Compound........ 363
Chilblaims..Sulphurous Acid in,.... 120
Chlor-alum, ................. 429
Treatment of,..........431–546

Chills. Chronic...Prescription for.... 184
Child's Remedy..Aconite, ........ 373
Chloride of Soda..In Burns and Ulcers,.................... 16
Sodium in Dysentery,....... 73
Of Ammonia..Uses of,...... 117
Of Zinc Paste,............. 359
Chlorate of Potass..In Dysentery,.. 124
In Abortion,................ 176
Is it a Parturifacient? By Dr. A. H. Brantley,........... 526
Chloroform..As a Febrifuge,....... 21
Death from,................ 24
Loss of Speech after,....... 25
Pro et con,................63–98
Internal use of, ........... 288
Ointment, .. .............. 358
Narcosis, ice in the Rectum for,................. 434
Elixer, for colic,........... 489
Chloral..Cebro-Spinal Meningitis,.. 23
Poisoning by,............... 49
In Hernia,................ 108
In Asthmatic Bronchitis.... 119
In Hooping Cough,........ 125
To Children,.............. 175
Palatable Formula for,..... 243
And Morphia in Tetanus,.. 246
In Strychnia Poisoning,.... 246
In Puerperal Mania,........ 251
In Puerperal Convulsions.. 251
In Epilepsy,............... 295
In Tetanus,.... ........... 296
In Singutus... ........... 300
In Rigid Os Uteri,.......... 301
In Obstetrics.............. 313
Purification of............ 369
In Midwifery............... 370
In Delirium Tremens...... 372
By Dr. T. S. Powell........ 398
In Intermittent Fever...... 432
In Enurisis................ 436
A Formula for.............. 599
Notes on.................. 615
Cholera Infantum..A case of....... 59
Bismuth in................ 308
By Dr. J. Branham......... 460
Hypophosphites in, by Dr. W. T. Taylor.............. 577
Bromide of Potass. in...... 534
Diet in.................... 537
A prescription for......... 597
Morphia, Hyphodermically, in...................... 75
Epidemics, Copper in...... 295
Portion for first symptoms.. 539
Turpentine a preventative of ..................... 561
Morbus .. Morphia, Hypodermically, in........... 343
Morbus..Treatment of..... 495
Morbus..Recini Collodion in...................... 605
Treatment of............. 664
Creosote in................ 667
Opium in................... 677
Morbus..Treatment of..... 664
Chloræthyl..A New Anæsthetic..... 254
Chlorosis..Presciption for.......... 303
Treatment of.............. 616
Chorea..Sulphate of Manganese in,. 71
Treatment of........... .. 76
Ether Spray in.........245–309
A prescription for.......... 357
Dr. West, on............... 597
Treated by Strychnia,..674–676
Chlorate of Potass. in Dysentery.... 660
Chloride of Zinc..Mitigated Stick.. 668
Cod-Liver Oil..Administration of... 670
And Iodide of Iron.... .... 682
Carbolized Gargled................ 661
Mixture for zymotic diseases 661
Calculi Biliary..A Remedy for...... 683
Chronic Inflammation of Bladder... 22
Dysentery..Nux Vomica in, 67
Citric Acid..In After pains......... 75
Cleaning Blankets................. 87
Cod-Liver Oil..Disguised.......... 257
Made palatable.........437–440
And Chloral............... 437
Coffee..As an Antidote............. 358
Colon..Flatulent Distention of...... 62
Collin's Disinfecting Powder........ 485
Colocynth..Prussian Tincture...... 312
Colic.............................. 108
Billious..A prescription for, 121
In Infants..Formulæ for... 185
Nux Vomica in............ 560
Colliquative Sweats .. Prescription for ..... ................ 182
Coma..By Dr. R. Battey............ 120
Compensation ........ ...... ..... 83
Copaiva..New mode of giving...... 407
Consumption..Digitalis in.......... 23
Coal Tar in. .............. 125
Condy's Fluid..For Wounds......... 107
Conium..Preparations of........... 252
In Mastitis............... 662
Constipation, Chronic..Therapeutics of....................... 50
Croup..Bromide of Potass. in....... 673
Cystalgia..Gelseminum in.......... 667
Chronic..Calabar Bean in.. 170
Pill for................... 535
Convulsions, Infantile..Turpentine in ..................... 304
Infantile..Caused by blows on the head............. 307
Treated by large doses of Quinine, by Dr. E. H. W. Hunter............... .. 645
Puerperal..Chloral Suppositories in................ 656
Corns..Cure for.................... 145
Cornea, Supuration of..Calabar Bean in ....................... 335
Cough..Mixtures .................. 242
Alum a Remdy for......... 16

Vapor of Slacking Lime in. 300
Local application in........ 315
Treatment of..........348-357
Glycerine Inhalation in..... 436
Corrosive Sublimate..For the Constitution.................. 247
Correspondence .................. 79
Crawford, Dr. G. G...Strangulated Hernia ................. 45
Uterine Catarrh.......... 107
Creosote..In Salivation............ 24
Cundurango ....................298-429
Cystitis..Sulphite of Soda in......... 181
Cystorrhœd..Oil of Erigeron in....... 560

**D**

Dahlberg's Tincture............... 612
Death from Chloroform............. 24
Of Dr. C. R. Harriss........ 628
Deafness..Treatment for .......... 481
Deception ........................ 147
Delirium Tremens..Chloral in...... 572
Solution of Ammonia in.... 430
Dentition..Bromide Potassium in... 177
Diabetes..Opium in................ 63
Treated by Carb. Ammonia, 297
Prescription for............ 307
Belladonna locally in....... 362
Sulphite of Soda in......... 488
Diet for.................... 604
Diarrhœa..Infants, due to Indigestion, Formula for......... 66
Of Children................. 66
Subnitrate of Bismuth in... 71
Infantile..Treatment of ... 121
Use of raw meat in........ 143
Treatment of............. 173
Sulphate of Copper in...... 174
Of Infants................. 178
Of Children............... 305
A Portion for.............. 362
Diarrhœa,......................... 366
Dr. Dugas Formula......... 367
A Mixture for............. 480
Arsenic in................ 483
Of Children............... 545
Chronic, Lugol's Solution in, 431
Chronic, Treatment of..... 441
Chronic .................. 594
Uremic ................... 600
Diaphoretic Powder............... 69
Diet of Parturient Women.......... 115
Difficult Micturition in Women.... 128
Digitalis..In consumption.......... 23
In Menorrhagia............ 74
In Heart Diseases......... 311
I fusing of............... 552
Dinner Pills...................... 132
Diptheretic Angina..Sulphur in..... 24
Diptheria..Treatment of........... 113
Local use of carbolic acid in, 118
Carbolic Acid in .......... 129
Etiology and Treatment of, 170
Prescription for........... 180
Cauterization in........... 240
A Remey for.............. 252
Treatment by Quinine..... 300
Ice, locally............... 355
Sesqui-Chloride of Iron and Glycerine in............ 480
Treatment of............. 544
Dislocation of Shoulder .. A New Method to Reduce....... 369
Reduction of... .......... 600
Dizziness..Cause and Prevention.................. 140
Dovers Powder..A substitute for... 69
Drake, Dr. W. G... Sloughing of Uterus................... 577
Dropsy Scarlatinal..By Croton Oil.. 121
Scarlatinal..Diaphoresis in, 478
A Prescription for......... 296
Dropsical Effusions................ 239
Dysentery, Chronic..Nux Vomica in, 67
Chronic, Terminating in Perforation..By Dr. I. W. Angel ...................... 580
Chloride of Sodium in...... 73
Chlorate of Potass in....... 124
Creosote in............... 128
Codea, Oxelate of Cerium in, 300
Morphia, Hypodermically... 313
Treated by Ipecac......... 314
Dr. Herberden's Treatment 366
Nitrate of Silver in......... 366
Its Treatmen..By Dr. W. T. Goldsmith ................. 411
Formula for............... 475
A New Remedy for Tenesmus of................... 548
Bismuth in................ 556
Dysmenorrhœa..Indian hemp in.... 121
Bromide Potass. in........ 125
Operation for.............. 256
Dr. Fenner's formula....... 365
Cannabis Indica........... 442
Prescription for........... 546
A New Remedy for........ 548
Dyspepsia..Nux Vomica for........ 122
From fermentation......... 126
A prescription for..127-143-292
Treated by raw meat....... 263
Arsenic in................ 301
Prescription for...... : .... 497
Morphia, hypodermically,... 498
Sulphite of Soda in........ 532
Excellent formulæ for.....: 563
Dyspeptics..Food for Nervous..... 141
Dandruff..A Remedy for........... 11
Dention Fever..Treatment of ..... 667
Diarrhœa..Wet Sheets in.......... 679
Chronic..Dr. J. Hale's Formula for................ 660
Chronic.................. 538
Diaphoretic..An efficient.......... 680
Digitalis..In hemorrhage of abortion 673
As a Remedy.............. 676
Diphtheria..Carbolized Gargle in.. 661
Carbolic Acid in........... 675
Distention of Bowels by Gas........ 660

**Dysentery**..Chlorate of Potass. in... 660
Chronic.. Chloride Ammonium in ... 666
Chronic ... 538
Chronic..A prescription for 683
**Dysmenorrhœa**..Dr. Hale's formula 657
**Dysmenorrhœa** ... 672
**Dyspepsia**..Dr. Hammond's formula 666
Of Pregnancy..A Remedy for ... 684

**E**

**Eczema**..Carbolic Acid in ... 68
Cod Liver Oil in ... 561
Chronic, Dr Barron's Treatment of ... 299
**Editorial** Correspondence ... 626
**Emmenagogue**..Valuable ... 72
Stimulating ... 62
**Epilepsy**..Prescription for ... 25
Bromide of Lithium ... 26
Treated by Chloral ... 295
Asafœtida in ... 296
Bromides in ... 597
And Enuresis ... 620
Morphia, hypodermically... 355
Strychnia in ... 498
From disease of ear ... 679
**Epistaxis**, Obstinate..Method for arresting ... 74
Monsels Salt in ... 248
**Epithelima**...Treated by Chlorate Potass ... 297
**Ergot**..In Retention of Urine ... 23
In Neuralgia ... 68
In Purpura ... 58
Antiplogistic..Value of... 372
In spinal congestion ... 678
**Ergotine**, hypodermically, in Aneurism ... 295
After amputation ... 244
**Eructations**..Sulphurous Acid in... 233
**Erysipelas**..Treatment of Infantile.. 70
Iron and quinine, locally... 174
A remedy for ... 602
Tr. Iron in ... 366
Quinine in ... 492
A formula for ... 681
**Extract** Fulton County Med. Society, 1
Proceeding Cincinnati Academy of Medicine ... 8
From letter of valued friend 34
**Exercise** before breakfast ... 90
**Exciting** causes to be avoided ... 141
**Extracts** from Correspondets ... 648
**Eye**..To remove particles from ... 146
**Earache**. Digitalis in ... 366
In children ... 657
**Eczema**..Treatment of ... 542
Oleum tiglii in ... 545
**Electrolysis**..In hydatid tumors ... 663
**Emmenagogue**...A new ... 306
**Emulsio Hydrocynata** ... 473
**Enemata**..By Dr. Robert Battey... 405
**Entropium**..A new operation for... 304
**Enurisis**..Chloral in ... 436
**Epileptic Mania**..A formula for... 668
**Epistoxis**...Tansey in ... 545
**Ether**..In nervous flatulence ... 305
**Exanthemic Liniment** ... 366

**F**

**Fainting Fits**..How to cure ... 86
**Fever**..Typhoid ... 65
**Felons**..Abortive Treatment ... 477
**Fœted Breath**..Cause and Remedy. 381
**Fish**..As an article of diet ... 142
**Fissure of Anus**..Tannic Acid in... 24
**Fistula** in Ano..Cure for without knife ... 313
**Flatulent** Distention of Colon ... 62
**Flatulence**..Ether in Nervous ... 305
Its Treatment ... 547
**Fibrous Growths**..Chloro-acetic acid in ... 678
**Fracture** of Clavicle..Dressing for.. 619
**Fulton** County Med. Society.. Extracts from ... 1-43-101

**G**

**Gangrene**..Senile ... 356
**Gangrenous** Vulvitis ... 612
**Gallaway**, Dr. C. B...On Treatment of Tetanus ... 589
**Gargle**, Carbolized..In Diptheria, &c. 661
**Gallactorrhœa** ... 361
**Gastralgia**..Dr. Hale's formula for.. 656
Treated by Electricity ... 676
**Gelseminium**..Incontinence of Urine 124
Sempervirens ... 129-327
Therapeutic effects of ... 249
Therapeutic Value of ... 608
**Georgia** Medical Society ... 328
**Glands** of Stomach ... 603
Neck..Suppuration of ... 606
**Gleet**..Carbolic Acid in ... 26
Bromide of Potassium in... 247
An Efficient Remedy in... 655
**Globe Flower**..By Dr. R. C. Word.. 523
**Glycerate** of Tannin..Dr Clymer... 12
Tar ... 302
Cream ... 561
**Goldsmith**, Dr. W. T.. Case Cerebro-Spinal Meningitis ... 3
Case Penetrating Wound of Abdomen ... 3
Case Hysterical Mania ... 43
Case Stranuglated Hernia.. 45
Treatment of Dysentery... 411
**Gonorrhœa**...Remedies for ... 21
Treated by Tannin and Glyerine ... 48
Glycerine of Tannin in ... 129
Extract Cannibis Indica in. 131
Treated by local means... 173
Permanganate of Potass. in, 251
Treatment of ... 486
Prescription for ... 538
Treatment of ... 562
Warm water Injections in.. 605

Spirits of Sandel wood in... 662
Gonorrhœal Peritonitis............ 487
Opthalmia ................ 487
Goodness better than Greatness.... 89
Granular Lids..Prescription, 124-301-309
Grant, Dr. W. T...Rattle-Snake poison and its Remedies.... 457
Greene, Dr. W. A...Hypodermic Administration of Medical Agents,........ . 159-227-286
Growth of Nails..A prognosis in Cerebral Paralysis....... 298
Gynœcological Medicine .. By Drs. Powell and Goldsmith, .................217-277-337

H

Hair Falling Off..Remedies for..... 489
Restoratives..Analysis of..
Wash..(Best).............. 663
Hartsen, Dr..Marriage of Consumptives .................... 53
Hay-Asthma..Quinine in........... 562
Headache, Obstinate..Nitrate Silver in...................... 122
Cannabis Indica in......... 315
Bromide Potass. and Gelseminium in.............. 543
Heart Disease...Ergot in........... 442
Digitalis in............563-676
Hemorrhage..Uterine.............. 68
Uterine..Turpentine in.... 244
Post-partum.............. 306
Tr. Erigeron in............ 488
After Abortion.....Digitalis in...................... 673
Uterine..Pressure in...... 678
Hemorrhoids..Prescription for...... 122
Carbolic Acid in........... 146
Calomel on............... 177
Prescription for............ 184
Arsenic in................ 497
Prescription for............ 541
Ointment for............. 539
Treatment for............ 593
Internal..Treatment of... . 606
Suppositories for.......... 663
Hemorrhoids ..................... 683
Hematuria Miasmatic.............. 120
Hemostatic..Tr. Erigeron as a..... 492
Hemicrania..Electricity in........ 540
Hemoptysis..Ergot in............. 556
A New Remedy for.. ..... 572
Hepititis, Chronic.. Prescription for 130
Suppurating .. By Muriate Ammonia.............. 303
Chloride Ammonium in.... 663
Hepatic Abscess..Chloride Ammonium in .............. 663
Hernia..Chloral in................. 108
Reduction by Morphia, hypodemically ............ 239
Reduction of............ 604
Hereditary Twin Bearing family...... 145
Herpes Zoster..Belladonna in...... 245
Collodion in............... 498
Hiccough..Persistent............... 132
Obstinate.................... 131
Obstinate, Chloral in....... 300
Hooping-Cough..Asafœtida in...... 16
Chesnut-leaves in......... 24
Prescription for............ 66
Remedy for............... 76
By Chloral ................ 125
Management of............ 177
Ioduret of Silver in........ 364
Prescription for............ 368
Chloral in................. 436
Cured by local application.. 473
Cimicifuga in............. 485
Tonka Bean in............. 497
Prescription for........... 537
Carbolate of Lime in....... 558
Sun-flower Seed in........ 661
A Remedy for............. 664
Honesty is the Best Policy......... 622
Horse Radish..Iodized Syrup of.... 68
Hospital Gangrene..Camphor in.... 366
How to Travel for Health.......... 143
Hydrate Chloral in Cerebro-Spinal Meningitis.............. 23
Chloral ................... 179
Hydrangea Aboresens.............. 173
Hydrocele..Radical cure for....... 358
Treatment of.............. 544
Hydrophobia..Potass. in........... 360
Hydrocepalus, Chronic............. 535
Infantile .................. 596
Conii in...................c. 681
Hypophosphites .. In Toothache of Pregnancy .............. 22
Hypophosphite Soda............... 146
Hypochondriasis..Strychnia in..... 75
Prescription in............ 182
Hypodermic Administration of Medical Agents.............. 159
Medication.............225-286
Injection of Ergot in Aneurism ................... 295
Doses of Morphia.......... 302
Syringe..Use of.......... 355
Medication ............... 438
Injection of Quinine........ 670
Hysteria..A Mixture for........... 476
A New Test for........... 438

I

Ice, to skin in excessive Menstruation .................... 76
Icterus, Infantile..Belladonna in... 74
Iodine Formula Improved.......127-363
As an application to wounds 364
Paint..A new............ 552
Gargle.................. 552
Iodoform......................... 254
Ointment ................. 314
As a Neurotic.............. 558

Injection..Starch as a Vehicle..... 671
Iodine in various diseases.......... 672
Iodide of Calcium................. 677
Iodide Ammonia preferred to Iodide Potass................ 437
Iodized Milk..................... 552
Iodo-Bromide of Calcium Comp .... 545
Improved Drink....... ........... 481
Incontinence of Urine..Belladonna in...................... 122
Gelseminum in.....122-146-246
Of old people..Iodine in.... 363
Management of........... 486
Indian Hemp..Menorragia and Dysmenorrhœa ............. 124
Indigestion, Functional..Treatment of..................... 493
Induction..Of Premature Labor.... 72
Infants..Bromide Potass. for....... 75
When to give food......... 143
A remedy for Chapped Skin, 430
Artificial Food for........ 433
Sleeplessness in........... 443
Infantile Erysipelas..Treatment of.. 70
Infancy..Mortality of.............. 329
Inflammation of Breast..Embrocation for................ 181
Influence of Abnormal Milk......... 361
Athletic Sports............ 261
Ingrowing Toe Nail................ 72
Insanity and Poor Diet............ 143
Epileptic..Castration for... 257
Ergot in....... ......... 682
Insomnia..Bromide Potass. and Ammonium ................ .. 359
Intermittent Fever..Strychnia in... 20
Iodide Potass. in.......125-174
Bromide Potass. in......... 240
Prescription for....307-309-365
Chloral in................. 432
Carbolic Acid in........... 439
A formula for............. 684
Intra-Uterine Medication........... 56
Intussusception..Warm water in... 304
Intestinal Obstructions............ 637
Inverted Toe-Nail..Dr. Pancoast's Treatment .............. 434
Is it proper for consumptives to marry 53
Itch..Treated by Balsam of Peru.... 61
Treatment for............. 250
Management of.........371-435
Treatment of............. 615
A Superior Remey for...... 662
Iron..Liquid Oxysulphate of........ 182
Effervescing Powders of.... 185

J

Jaundice..Chion. vir. Old Man's Beard ni................. 359

K

Kidney Disease..Vomiting a Symptom of.................... 672
Kali-Kuki..A New Tonic......... 252
Kollock, Dr. G. J...On Neuralgia of Uterus.................. 528

L

Labor..Induction of Premature..... 72
Lacteal Secretion..Arrested by Belladonna................. 73
Lactate of Iron.................. 361
Laxative and Alterative Mixture.... 132
Leeches and Mustard.............. 251
Letter from Cincinnati............. 586
Leucorrhœa..Remarks on......... 111
Iodine Injection in......... 131
Lactate of Iron in....... .. 170
Bromide of Potass. in..181-247
Carbolic Acid in........... 445
Arsenic in................. 483
Lichen..Aconite in................ 127
Liniment..For Neuralgia........... 65
A Stimulative............ 555
Dr. Hodges............... 665
Lithium, Bromide of..In Epelepsy.. 26
Uses of .................. 60
Liquor Potassæ..In Stranguary..... 23
Sedativus..Formula for.... 666
Liver..For Food.................. 143
Lobelia in Children................ 316
Locomotor Ataxia..Phosporous in.. 559
Look to your Pumps and Water pipes...... ............ 88
Loss of Speech after Chloroform.... 25
Lowndes County (Miss.) Medical Association.............530-652
Lumbago and Sciatica..Prescription for ................... 126
Rheumatic................ 183
Treatmet of................ 302
Poultices in............... 309
Lupulin..As a Sedative............ 128
Lycoperdon..A Styptic. .......... 559
Lymphatics.Strumous Enlargement of..................... 69

M

Macon Med. Ass........235-269-271-581
Magnesia Sulphate..Made palatable 557
Mammary Abscess....... ........ 543
Inflammation..Conium in.. 662
Management of Placenta........ ... 15
Of Perineum in labor....... 117
Mania Hysterical.. By Dr. W. T. Goldsmith............ 43
Bromide Potass. and Cannabis, Indica in............ 561
Epileptic..A Formula for... 668
Marigold..In Wounds............ 71
Measles..Prophylaxis of.......... 69
Management of.... ...... 466
Medical Society District Columbia.. 42
Of Virginia............... 50
Medication..Inta-uterine .... ..... 5
Menorrhagia..Bromide Ammonium. 21
Digitalis in.............. 24
Indian Pemp in.......... 171
Carbonate of Iron in....... 183

Menstration, Excessive..Ice to skin for.................... 76
Mettaurer's Aperient Solution...... 122
Mettorrhagia..Ergotine, hypodermically, in................. 559
Migrain..One Side Headache.. .... 146
Mixture of Quinine and Sulpho-Carbolate of Sodium........ 662
Morphia..A useful Prescription for. 250
Music for Invalids................ 90
Myalgia, Pain of Muscles..Muriate Ammonia in............. 192

N

Nasal Catarrh..A Snuff for....... 667
National University................ 203
Nettle Rash..Bromide Potass. in.... 310
Nervous Affection of Abdominal Organs of Women..A prescription for.............. 67
Nervous Affections.. Muriate Ammonia in................ 241
Flatulence..Ether in....... 305
Neuralgia Liniment................ 65
Prescription for.............. 67
Ergot in.................. 68
Sulphate of Nickel in...... 71
Hypodermic use of Morph. in...................... 131
Intercostal of Sucking Women, treated by Muriate Ammonia............... 192
Morphia Collodion in....... 242
Cardiac.................... 246
Formula for................ 296
Oil Pepperment in.......... 304
With Shingles..A remedy for...................... 309
Citrate of Caffien in........ 359
Ergot and Ver. Viridi in.... 442
Electricity in............. 492
Ovarian.................... 496
Of Uterus .. By Dr. G. J. Kollock................. 528
Ovarian..A formula for.... 552
Dr. Hale's formula for...... 668
Neuralgic Pill.................... 128
Nevus..Treatment of.............. 67
New Hypodermic Syringe.......... 275
Night Sweats..Treatment of....... 560
Nipple..Nitrate of Lead in Sore.... 25
Retracted................ 61
Remedy for Sore.......... 131
Treatment of Sore.......... 469
Inflamed.................. 474
Wash for Sore............. 546
Salve for Chapped.......... 553
Nitrate of Silver in Orchitis......... 21
Of Silver in obstinate headache...................... 122
Of Silver in Vomiting of Pregnancy.............. 124
Of Amyl................... 616
Nonat, Dr... Use of Arsenic in Phthisis................. 14
Nux Vomica in Chronic Dysentery.. 67
In Dyspepsia and hypochondria...................... 122
In Billiousness.............. 296
In Catarrh of Stomach..... 314

O

Oakum and Carbolic Acid as an Antiseptic Dressing........ 653
Obstinate Vomiting..Case of....... 56
Obesity..Cure for................. 146
Oil of Peppermint as an anæsthetic.. 254
Ointment for Old Sores............ 478
Oleum Terebinthini................ 655
Onychia..Nitrate of Lead in........ 494
Opium..In Diabetes............... 63
In Tetanus................. 64
And Belladonna antagonistic....................... 305
Opacity of Conea.................. 184
Ophthalmia..Gonorrhœal........... 487
Orchitis..Nitrate of Silver in....... 21
Digitalis in................ 176
Treatment of............... 265
Originator of Pathological Anatomy, 145
Ottorhœa..For relief of pain of...... 299
Chronic..A remedy for..... 549
Sulpho-Carbotate Zinc in... 662
Our Nashville Correspondent...... 10
Journal................... 27
Journal..First number..... 34
Our Advertising Friends........... 37
Exchanges.................. 40
New Deparment............ 77
Carolina Correspondent.... 187
Journal.................... 382
Associate Editors.......... 446
Corresponding Editors..... 501
Platform.................... 564
Owen, Dr. W. G...Coffee in Strangulated Hernia.............. 45
Ovaritis............................ 658
Oxalate of Cerium in Dyspeptic Vomiting........................ 65
Ortorrhœa..Treated by Spirit of Wine.................. 672
Opium..Verat. Viridi an antidote in. 675

P

Paralysis..Local Prescription for... 76
Infantile..Treatment of.... 478
Of Bladder..Perchloride of Iron in................ 664
Palpitation of Heart..Gelseminum in 666
Peaslee, Prof. E. R...Intra-Uterine Medication................. 56
Penetrating Wounds of Abdomen... 3
Pepsine..Uses of................. 71
Scheffer's Preparation of. 488
Permanganate of Potass. in Female Disease................. 12
Permanganate of Potass............ 496
Permanent Contraction of Limb Cured..By Atrophia, hypodermically............ 243
Perishing for lack of knowledge.... 86

Perspiration ........................ 89
Formula for excessive..... 536
Ergot in excessive......... 543
Peritonitis; Meretricum............. 477
Pericarditis..Verat. Viridi in...... 556
Perchloride of Iron in Post-Partum Hemorrhage ............ 22
Pharynx..Irrigation of............. 611
Phthsis..Coal Tar in............... 126
Glycerine in................ 124
Carbolic Acid in........... 362
Saponified Cod-Liver Oil in 475
Prescription for Incipient.. 490
A formula for.............. 664
Phlegmasia Dolens .. Treated by Opium................... 174
Iron in ..................... 614
Phosphorus Pill.................... 539
An antidote for poisoning of 556
Pill, Substitute for Compound....... 479
Pinckney, Dr. Chas., Reports Fulton County Med. Soeiety1–43–101
Case of Penetrating Wound of Abdomen.............. 3
Piles, Suppositories for............. 663
Pinus, Canadensis, Ulcers Os Uteri. 661
Placenta Previa, by Dr. E. J. Roach 46
Plaster, Poor Man's................. 664
Pneumonitis, Carbonate of Ammonia in ..................... 178
Arnica in.................. 263
Catarrhal of infants treated with Carb. Ammonia....... 354
Digitalis in................ 362
Infantile, Prescription for.. 491
Pneumothorax, Aspiration in...... 614
Post-Partum hemorrhage Perchloride of Iron in.............. 22
Poisoning by Chloral............... 49
Polypus of Nose, A cure for........ 184
Poultices in acute Lumbago......... 309
Poison Oak. A remedy for......564–603
Powell, Dr. T. S., Case of Spontaneus virsion.............. 1
Case shoulder presentation 45
Cases Cerebro-Spinal Meningitis.................. 101
On Chloral................. 398
Potassium, New use for.......... . 556
Pressure to Uterus, in lingering labor 112
Pregnancy. Hypophosphites in Toothache of.................. 22
Carbolic Acid in Sickness of 23
Vomiting of............... 24
Duration of................ 144
Salivation of.............. 192
Tubal...................... 237
Possible duration of........ 247
Vomiting of................ 357
Sickness of, Its Cause and Treatment ............... 468
Phosphate of Lime in Sickness of.................. 475
Premature Labor, Induction of..... 72
Proceedings Chicago Med. Society.. 4
Macon Med. Convention..374–388
Professional Unity.................. 91
Brotherhood ................ 193
Remuneration............. 209
Prophylaxis of Scarlet Fever and Measles ................ 69
Prophylamin in Rheumatism....298–474
In Rheumatism of Uterus..
Prolapsus Uteri, Curative effect of Pregnancy................ 306
Prostrate Gland, Treatment of inflammation of............ 441
Prostritis, Chronic, Arsenic in...... 497
Prurigo, Aconite in................. 127
Iodoform Ointment in...... 609
Pruritus, Prescription for........... 130
Carbolic Acid in............ 255
Vulvæ, Remedy for......... 687
Psorias, Cured by Iodide Potass..... 440
Copaiva ...................... 473
Puerperal Convulsions, Bromide Potass. in................ 22
Chloral in.................. 173
Puerperal............................ 251
Veratum Vir. in.........398–440
Chloral Suppositories in.... 656
Chloro for, by Stomach in.. 660
Chloroform and Chloral in. 662
Puerperal Mania, Chloral in......... 251
Treatment of............... 618
Prostration, Turpentine in.. 496
Purpuria, Ergot in................. 68
Of Children, Ergot in....... 250
Hemorrhagica. Turpentine in ......................... 356
Pulmonary Consumption, Treatment of ....................... 18
Carbolic Acid in............ 118
Pulse, The........................... 145
Pyæmia, Sulphites in................ 253
Treatment of............... 479
Pyrosis, Sulphurous Acid in........ 74
Phthsis Pulmonalis, A Formula for. 683
Placenta Previa..................... 674
Pregnancy, Vomiting of, cured by position ................. 676
Pruritus Vulvæ, Treatment of....... 680

### Q

Quassia, per Surgical Dressing...... 611
Quinine, Substitute for............. 60
To Disguise taste of........ 433
Muriate of................ 437
An Essay on............... 463
Pills......................... 472
Formula for giving......... 485
Biscuits .................... 486
Experiments with.......... 604
In large doses in convulsions of children, by Dr. E. H. W. Hunter ............. 645
Quinine, Hypodemic Injection of... 670

R

Radical Cure for Artificial Anus.... 48
Rattle-Snake Poison and its remedies By Dr. W. T. Grant...... 457
Raw Meat in Disease.............. 352
Red Ink........................... 610
Remedy for Dandruff................ 11
For Poison Oak............. 656
Remedies for Gonorrhœa............ 21
Remove Stains in Silver............ 366
Retention of Urine................. 23
Ice in Rectum for......... 598
Retracted Nipple.................. 61
Rheumatism, Permanganate of Potass. in.................. 75
Acute, Lime in............ 184
Prophylamin in..298–474
Treatment of...... 249
A Liniment for..... 477
Oil of Horse-chesnut in.............. 535
A Sure Cure for.... 610
Prophylamin in Urine...... 663
Chronic, Prescription for, 127–310
Diagnosis between Neuralgia and...... 315
Muscular, Remedy for..... 495
Rice, Uses of..................... 499
Rigid Os Uteri, Lobelia for......... 73
Chloral in................ 309
Hydrochlorate apomorphia in....................... 305
Remedy for............... 436
Gelseminum in............ 665
Ring-worm, Remedy for........... 24
Chromic Acid in........46–535
Roach, Dr. E. J., Case Placenta Previa......................... 45
Rubeold, Carbolic Acid in.......... 74
Rheumatism, Acute, Treated by Nitrate Silver, locally...... 681

S

Salivation, Creasote in............. 24
Of Pregnancy............. 192
A Wash for............... 476
Sambucus Canadensis, in Albuminaria ..................... 122
Santonine and Podophyllin......... 71
for Ascarides............. 73
Solution of............... 433
Scarlet Fever ..................... 20
Pathology and Treatment of 64
Propholaxsis of........... 69
Carbolic Acid in.......... 297
Ice locally for............ 352
Iron in.................. 612
Prevention of............ 682
Scarlatinæ Dropsey................ 121
Science and Peoce................. 94
Sciatica prescription for............ 126
Treatment of.............. 175
Treatment of.............. 358
Scrofulous diseases, treatment of.... 357
Scutelaria, therapeutic value of..... 537
Senile Gangrene, Choral in......... 543
Sewing Machines and health....... 90
Shall Medical Societies enforce their laws................... 199
Shoulder presentation, by Dr. T. S. Powell................... 45
Tip pain in hepatic diseases 51
Sickness of pregnancy, Carbolic Acid in....................... 23
Cause and treatment....... 468
Phosphate of lime in....... 475
Skin Diseases...................... 125
Grafting.................. 168
Affections................ 255
Diseases, purgatives in..... 306
Alkalies in............... 472
Sleeplessness, a remedy for......... 24
During fever.............. 364
In infants................ 443
Sleep, position of head during....... 144
Small-pox, to prevent pitting in..... 430
New treatment for......... 541
Hemorrhagica............. 617
A new treatment for....... 673
Hypophosphate of Soda in... 683
Snake-bite, Carbolic Acid in......... 23
Cure for.................. 532
Poisoning, antidote for.... 595
Solution for Opthalmoscopic investigation........................ 130
Soda Mint......................... 432
Sodium Sulpho, Carbolate.......... 497
And Quinine.............. 662
Sore Nipples, Nitrate of lead in..... 25
Spermatorrhœa, Bromide of Iron in 308
Belladonna in..... 431, 447, 594
Speaking without a tongue......... 248
Sprains, New treatment for......... 185
Liniment for.............. 362
Spinal Congestions, Ergot in........ 678
Spongio, piline.................... 312
Sponge Tents, how to made easily... 536
Staphisagria, Lice, remedy for..... 310
Strangulated Hernia............... 8
Cases, by Drs. Crawford, Goldsmith and Owen.... 45
Stranguary, liquor potassæ in...... 23
Strychnine in intermittent fever.... 20
in hypochondriasis.......... 75
Detection of, etc.......... 132
Poisoning, Chloral in....... 249
Antidote for.............. 480
Starch, as an injection............. 671
Strumous, enlargement of lymphatics 69
Stricture, continuous dilitation in... 257
Urethral............. 361–487
Styptic, a new..................... 545
Colloid................... 368
Sulphur in diptheritic angena....... 24
Substitute for Dovers Powders...... 69
Sulphate of Soda.................. 50
In tenia capetis............ 61
Sulphurous acid in Pyrosis......... 74

Sun flower, virtues of.............. 142
Sun-stroke, treatment of............ 555
Suppressed Bronchial Secretion.... 76
Suppression of Urine.............. 181
Suppuration, Sulphate of Iron in.... 369
Syphilis, prescrip. for Sec. and tertiary.................... 26
By Dr. F. T. Bumstead...... 669
Use of Sarsaparilla in... .. 70
Incubation period.......... 238
Secondary, prescrip. for.... 307
Tertiary, Bromide of Iron in 312
Calomel injection in........ 434
Sulphates in....... ...... 489
Treatment of............. 492
Iodoform in.... .......... 540
Creosote in............... 542
In children............... 542
Syphiletic, Ulceration of throat, Sulphurous Acid in......... 127
Pneumonia................ 128
Infection from kissing...... 144
Sloughing Sore Throat..... 171
White Swelling............ 183
Syphilitic Ulcers, prescrip. for...... 304
Sycosis, treatment of.............. 125

T

Tannic Acid, in fisures of Anus.... 24
Tannin and Glycerin in Gonorrhœa.. 48
Tape Worm, treatment for.......... 69
Expelled by Bromide potash.................... 301
Ether for................. 559
Taylor, Dr. W. T., Hypophosphites in Cholera Infantum..... 517
Tenea Capetis, Sulphite Soda in.... 61
Formula for........... 534-666
Tetanus, large doses of opium in... 54
Calabar Bean in........... 73
Chloral and Morphia in.... 246
Treated by Chloral........ 296
Neonatorum, Chloral in.... 436
Treated by Calabar Bean and Chloral, by Dr. C. B. Galloway ................ 589
Testicles, Ice in affections of....... 492
Teething, Bromide Potass, in....... 555
Therapeutic use of Arsenic in phthsis 74
Of Chronic Constipation.... 50
The American Med. Association..... 30
Mass. Med. Society........... 32
Atlanta Med. College & its Apologists...................... 384
Truth plainly told................. 574
Thraldrom of Fashion.............. 38
Things not generally known........ 247
Thomas, Prof. J. G., letter from..... 186
Throat, to examine the............ 604
Tight Lacing....................... 88
Toe-Nail, treatment of in growing... 72
Tonsils, gargle for ulceration of...... 132
Enlarged, treatment of..437-538-598
Tongue, Dr. J. M. Scudder on..... 658
Tonic, a favorite remedy........... 178
Tonsilitis, treatment of............ 184
Belladonna in.... ....... 481
Carbolized Gargle in........ 661
Toothache, of pregnancy, hypophosphites in............... 22
A remedy for...... 145-303-373
Chloral in.............. .. 611
Tooth wash....................... 141
To preserve Anatomical and Pathological Specimens.......... 597
Torticollis, operation for........... 487
Treatment or Ulceration, stomach and Cancrum Oris. ....... 16
Pulmonary Consumption... 18
Trismus Neonatorum, Calabar Bean in....................... 246
Nascentium............... 602
Tumors, Subcuteaneous removal of without hemorrhage....... 373
Mitchell's process.......... 606
Phanton ..... ............ 609
Diagnosis of phanton...... 659
Hydated, treated by electrolysis .................... 663
Turpentine, therapeutic action of.................. 436
Tympanitic, Distention, cold water for..................... 367
Tympanitis, relief of by puncture... 372
Puncture in....... ....... 557
Electricity in.............. 660
Typhoid Fever..................... 65
Belladonna in.......... 239-431
Sulphurous Acid in........ 245
Strychnia in............... 310
Saliceni in.... ........ 355-440
Glycerine in............... 551
Typhus Fever, Belladonna in....... 350
Tape-Worm, treatment for......... 671
The close of the year.............. 687

U

Ulcers, Chloride of Soda in......... 16
Iodoform in............... 182
Indolent prescription for.... 185
Syphelitic " ....... 304
Varicose, remedy for... 312-596
Gangrenous,............... 358
Carbolated Cerate in Varicose ...................... 475
Chronic, prescription for.... 486
Of leg, treatment of........ 490
Os Uteri.................. 611
Chronic, a lotion for........ 661
Os Uteri, Pinus Canadensis in....................... 661
Ulceration of Stomach, treatment of 16
Of tonsils, a gargle for...... 132
Opium in................. 471
Urine, Ergot in retention of........ 23
Retention of, hysterical.... 144
Suppression of............ 181

Urine, In continence of............. 255
Retention of, Ice in rectum for..................... 598
Urinary Disease, perchloride of Iron 180
Uses of Bromide of Lithium........ 60
Uterine, hemorrhage,........... 68–126
Catarrh, permanganate potass. in................... 107
Carbolic acid in........... 123
Affections, Arsenic in...... 124
Fibroias, Bromide potass in 368
Affections, ergot hyperdermically in............... 430
Diseases, bebeerina in..... 497
Uterus, Arsenic for enlarged cervix of...................... 483
Bebeerina in chronic inflammation of............... 548
Case of sloughing by Dr. W. G. Drake................ 577
Ulcerations, Oleum Terebinth. in... 678
Ulcers Serpig. Syphiliticum, a formula for................ 684
Uterine hemorrhage, pressure in.... 678
Uterine Epithetioma, chlorate of potass. in.................. 661

**V**

Vaginitis, Glycerate of Tannin in.... 491
Varicose Ulcers, Remedy for. 312–475–596
Viens, treatment of........ 441
Vermifuge......................... 71
Ventilation........................ 143
Venerial Vegitation, Treatment of... 364
Velpeau's Mixture for Diarrhœa.... 473
New Caustic............. 369
Vesico-Vaginal Fistulo, Treatment of 567
Victory........................... 265
Vomiting, Oxalate of Cerium in Dyspeptic................... 65
Obstinate.................... 66
Of Pregnancy............. 124
Sulphurous Acid in........ 239
Black, Oxide of Mercury in 357
Pepsin in.. ............... 534
A Prescription for......... 541
Raw Meat in............... 543
Columbo and Phosphate of Lime in................. 560
New mode of inducing..... 358
Volitile Liniment, an elegent formula 472
Vesication, a rapid mode of inducing 682
Vomiting, a prominent feature in Kidney Disease......... 672
Of Pregnancy cured by position .... ............... 676

**W**

Warts, a cure for................. 146
Remedies for.............. 603
Wakefulness, phosphorus for....... 196
Water, drinking in hot weather..... 90
Williams, Dr. A. M., permanganate potass. in female diseases 13
Wounds, dressed with tin-foil...... 435
Turpentine in............. 537
New treatment of......... 555
Word, Dr. R. C., in Globe-flower,.... 523

**Z**

Zinc, Sulpho-Carbolate of.......... 444
Zinci, Sulphatis et chlorate potass. A valuable combination of 541
Zymotic Diseases, a carbolized mixture for................. 661

### OUR EXCHANGES.

We return our heartfelt thanks—yea, more than thanks, our gratitude—to those gentlemen who have so kindly forwarded us their periodicals in advance of the publication of the COMPANION. We shall keep, ever fresh in mind, the fraternal kindness so generously extended to us and our enterprise.

We, therefore, with the greatest pleasure, acknowledge the reception of the following list of Medical Journals sent us. They will be found to embrace some of the best, most thorough, and most distinguished of the medical periodicals, published on the continent. Several others have not been received that we were informed would be sent, and some too late to be noticed.

*The Medical Times.*—A semi-monthly Journal, published by the well-known Publishing House, J. B. Lippincott & Co., 715 and 717 Market Street, Philadelphia; an establishment with which we expect to make our readers as familiar as children are with household toys. The Times is a journal of very great value, and should have an extensive circulation. Terms $4 in advance.

*The Medical and Surgical Reporter.*—A Weekly Journal, published in Philadelphia. Edited by S. W. Butler. M. D., D. G. Brinton, M. D. Terms—Five Dollars per annum, in advance. This is a very neat and exceedingly valuable Journal. We heartily wish it great success. Every wide-awake practitioner should be a subscriber to the "Reporter."

*The Dental Cosmos*, published in Philadelphia, by Mess. Jones & White. Edited by Drs. J. D. White, J. H. McQuillen and Geo. J. Zeigler. It contains much that is valuable to the general practitioner of medicine. To the Dentist we would say it is invaluable. Price, $2,50 a year, in advance.

*Chicago Medical Examiner.*—A Monthly Journal, of 60 pages. Edited by N. S. Davis, M. D., a polished gentleman and able medical writer. His Journal sustains his reputation as such. Price $3,00 in advance.

*The Cincinnati Lancet & Observer.*—Published monthly. Price 3,00 per annum. E. B. Stevens, M. D., Editor. J. A. Murphy, M. D., W. H. Mussey, M. D., E. Williams, M. D. Associate

Editors. This is a valuable Journal. Every member of the Profession who desires to keep up with its progress should take it.

*The Medical News and Library.*—A valuable monthly pamphlet, of 24 pages. Published by Henry C. Lea, Philadelphia, Pa. the great book publisher, about whom we expect to say a great deal in the future. This energetic publisher proposes to furnish, free of postage, a copy of Prof. W. B. Carpenter's Prize Essay on the use and abuse of Alcoholic Liquors in Health and Disease, to any gentleman who will furnish him a complete list of the Physicians, Dentists and Druggists of his County. We would suggest to the Temperance organizations of the South to fall in with this proposition.

*Boston Medical and Surgical Journal.*—Published every Thursday of each week*:* Francis H. Brown, M. D. Editor; H. H. A. Beach, M. D., Assistant Editor. Price, $3 00 per annum in advance. This is one of our most welcome exchanges. Long may it live, and never fail to pay us its weekly visits.

*Journal of the Gyneocological Society of Boston:* Published monthly, by James Campbell, 18 Tremont St., Boston: L. W. Schmidt, New York; and Leipzig, Germany. Edited by: Winslow Lewis, M. D., Horatio R. Storer, M. D., Geo. H. Bixby, M. D. This Journal is "devoted to the advancement of the knowledge of the diseases of women," and is an indispensable periodical to every Physician desirous of being up to the times in regard to the diseases to which it is specially devoted. It should be in the hands of every Physician. Price $5 00 per annum in advance.

*Nashville Journal of Medicine and Surgery:* W. K. Bowling, M. D., W. L. Nichol, M. D., Editors and Proprietors: Paul F. Eve, M. D., W. T. Briggs, Associate Editors. This Journal maintains its old reputation, and is one of our best exchanges. The editorial staff, for talent and distinguished merit, is not surpassed by any medical Journal in the country. Published at Nashville, Tenn. Terms, $3 00 per annum, in advance.

*The Medical Record, a Semi-Monthly Journal of Medicine and Surgery:* Geo. F. Shrady, A. M., M. D., Editor: Published on the 1st and 15th of each month, by Wm. Wood & Co., 61 Walker St. New York. This is a first class medical Journal, ably edited, and replete with the best medical literature of the day. It should have an extensive Southern circulation. Terms, $4 00 per annum, in advance.

---

### AN APOLOGY.

The first number of our journal has been delayed in its appearance, at the proper time, from a combination of circumstances which usually attends the issuing of the first number of any periodical. This we exceedingly regret. In this instance the Editors are responsible, from the fact that they did not know the exact time or the amount of matter to be placed in the hands of the publisher. In future the COMPANION will not be delayed from like causes, but will be issued promptly on the 15th day of each month.

